# Artificial intelligence-guided detection of under-recognised cardiomyopathies on point-of-care cardiac ultrasonography: a multicentre study

**DOI:** 10.1016/S2589-7500(24)00249-8

**Published:** 2025-02

**Authors:** Evangelos K Oikonomou, Akhil Vaid, Gregory Holste, Andreas Coppi, Robert L McNamara, Cristiana Baloescu, Harlan M Krumholz, Zhangyang Wang, Donald J Apakama, Girish N Nadkarni, Rohan Khera

**Affiliations:** Section of Cardiovascular Medicine, Department of Internal Medicine (E K Oikonomou MD, R L McNamara MD, H M Krumholz MD, R Khera MD), Department of Emergency Medicine (C Baloescu MD), Department of Biomedical Informatics and Data Science (R Khera), and Cardiovascular Data Science (CarDS) Lab (E K Oikonomou, G Holste BA, R Khera), Yale School of Medicine, New Haven, CT, USA; The Charles Bronfman Institute for Personalized Medicine, Icahn School of Medicine at Mount Sinai, New York, NY, USA (A Vaid MD, G N Nadkarni MD); Division of Data Driven and Digital Medicine, Department of Medicine, Icahn School of Medicine at Mount Sinai, New York, NY, USA (A Vaid, G N Nadkarni); Department of Emergency Medicine, Icahn School of Medicine at Mount Sinai, New York, NY, USA (D J Apakama MD); Department of Electrical and Computer Engineering, The University of Texas at Austin, Austin, TX, USA (G Holste, Z Wang PhD); Center for Outcomes Research and Evaluation, Yale–New Haven Hospital, New Haven, CT, USA (A Coppi PhD, H M Krumholz, R Khera); Section of Health Informatics, Department of Biostatistics, Yale School of Public Health, New Haven, CT, USA (R Khera)

## Abstract

**Background:**

Point-of-care ultrasonography (POCUS) enables cardiac imaging at the bedside and in communities but is limited by abbreviated protocols and variation in quality. We aimed to develop and test artificial intelligence (AI) models to screen for under-diagnosed cardiomyopathies from cardiac POCUS.

**Methods:**

In a development set of 290 245 transthoracic echocardiographic videos across the Yale–New Haven Health System (YNHHS), we used augmentation approaches, and a customised loss function weighted for view quality to derive a POCUS-adapted, multi-label, video-based convolutional neural network that discriminates hypertrophic cardiomyopathy and transthyretin amyloid cardiomyopathy from controls without known disease. We evaluated the model across independent, internal, and external, retrospective cohorts of individuals undergoing cardiac POCUS across YNHHS and the Mount Sinai Health System (MSHS) emergency departments (between 2012 and 2024) to prioritise key views and validate the diagnostic and prognostic performance of single-view screening protocols.

**Findings:**

Between Nov 1, 2023, and March 28, 2024, we identified 33 127 patients (mean age 58·9 [SD 20·5] years, 17 276 [52·2%] were female, 14 923 [45·0%] were male, and for 928 [2·8%] sex was recorded as unknown) at YNHHS and 5624 patients (mean age 56·0 [20·5] years, 1953 [34·7%] were female, 2470 [43·9%] were male, and for 1201 [21·4%] sex was recorded as unknown) at MSHS with 78 054 and 13 796 eligible cardiac POCUS videos, respectively. AI deployed to single-view POCUS videos successfully discriminated hypertrophic cardiomyopathy (eg, area under the receiver operating characteristic curve 0·903 [95% CI 0·795–0·981] in YNHHS; 0·890 [0·839–0·938] in MSHS for apical-4-chamber acquisitions) and transthyretin amyloid cardiomyopathy (0·907 [0·874–0·932] in YNHHS; 0·972 [0·959–0·983] in MSHS for parasternal acquisitions). In YNHHS, 40 (58%) of 69 hypertrophic cardiomyopathy cases and 22 (46%) of 48 transthyretin amyloid cardiomyopathy cases would have had a positive screen by AI-POCUS at a median of 2·1 (IQR 0·9–4·5) years and 1·9 (0·6–3·5) years before diagnosis. Moreover, among 25 261 participants without known cardiomyopathy followed up over a median of 2·8 (1·2–6·4) years, AI-POCUS probabilities in the highest (*vs* lowest) quintile for hypertrophic cardiomyopathy and transthyretin amyloid cardiomyopathy conferred a 17% (adjusted hazard ratio 1·17, 95% CI 1·06–1·29; p=0·0022) and 32% (1·39, 1·19–1·46; p<0·0001) higher adjusted mortality risk, respectively.

**Interpretation:**

We developed and validated an AI framework that enables scalable, opportunistic screening of under-recognised cardiomyopathies through simple POCUS acquisitions.

**Funding:**

National Heart, Lung, and Blood Institute, Doris Duke Charitable Foundation, and BridgeBio.

## Introduction

Point-of-care ultrasonography (POCUS) enables focused cardiac evaluation across communities, outpatient clinics, emergency departments, and inpatient facilities.^1^ In contrast to standard transthoracic echocardiography, POCUS studies are acquired in busy clinical settings following abbreviated protocols. Consequently, image quality and the information yield are limited due to environmental factors (eg, equipment and time constraints), patient characteristics (eg, an inability to reposition themselves, distress, individual disease states, and anatomy), and operator experience (eg, acquisition by novices or trainees).^2^ Therefore, POCUS videos are rarely used beyond addressing acute questions.

With the expanding use of handheld technology that can assist novice operators in acquiring cardiac views,^3^ POCUS imaging offers an opportunity for scalable cardiovascular screening. In particular, this ability of POCUS to facilitate accessible and efficient imaging could be of value in the screening of under-diagnosed cardiomyopathies, such as hypertrophic cardiomyopathy or transthyretin amyloid cardiomyopathy. Both conditions are associated with increased morbidity and mortality and benefit from early recognition^4,5^ and initiation of disease-modifying therapies.^6–10^ Unfortunately, their detection remains challenging due to a long pre-symptomatic course, overlapping clinical phenotypes, and the reliance on advanced multimodality imaging.^4,5,11^ Although hyper trophic cardiomyopathy is considered the most common cardiomyopathy with a prevalence of about one in 200 individuals,^11^ the reported estimates for transthyretin amyloid cardiomyopathy^12^ likely under estimate its true prevalence, with non-invasive imaging studies showing high prevalence among individuals with aortic stenosis, or heart failure with preserved ejection fraction, ranging from 1% to 13%.^13–15^ Despite that, only a minority (10–20%) of hypertrophic cardiomyopathy or transthyretin amyloid cardiomyopathy cases are identified clinically,^16,17^ whereas the need for multi-modality imaging could further exacerbate diagnostic disparities.^18^ Although transthoracic echocardiographic imaging has benefited substantially from advances in computer vision and medical artificial intelligence (AI),^19–23^ these algorithms are built for use with videos acquired by expert technicians. Furthermore, while access to transthoracic echocardiograms remains limited and prone to referral and selection bias,^18^ POCUS studies are increasingly done across high-resource and low-resource settings,^24^ and represent an untapped yet accessible resource for opportunistic screening.

Here, we propose and implement a framework for POCUS-adapted video-based AI models, showing their ability to efficiently detect under-diagnosed cardiomyopathies from real-world POCUS studies acquired over a decade across the emergency departments of two large and diverse health systems. Our approach incorporates a range of natural and synthetic augmentation methods to simulate off-axis acquisitions from variable views, thus enabling downstream inference from limited POCUS protocols. Building on this approach, we further aimed to assess the diagnostic and prognostic potential of AI-POCUS, offering insights into missed or delayed diagnosis.

## Methods

### Study overview and objectives

**Figure 1:**
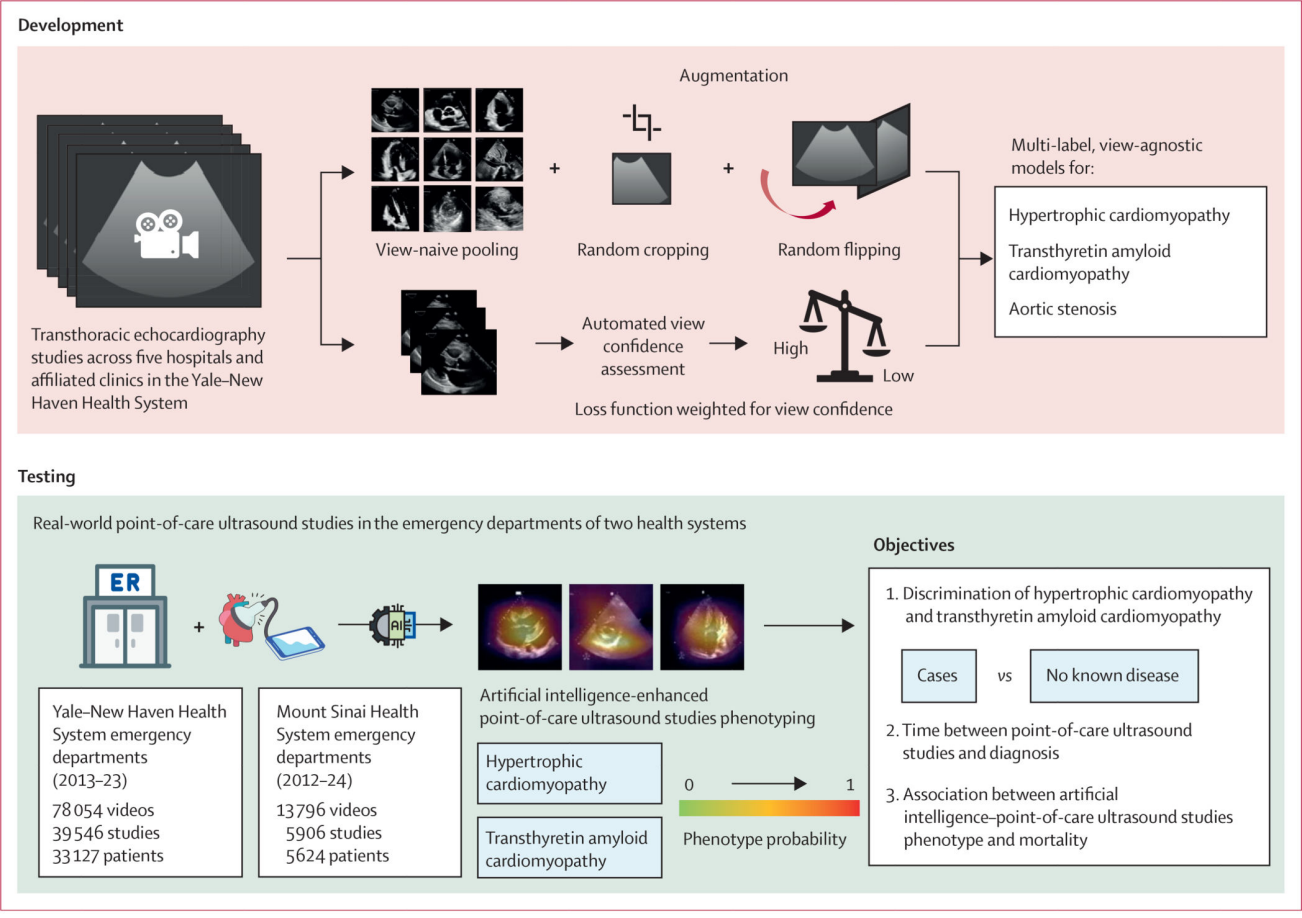
Study overview

The overall objective was to develop and test video-based deep learning algorithms for the efficient diagnosis of hypertrophic cardiomyopathy and transthyretin amyloid cardiomyopathy on POCUS. First, we used a library of transthoracic echocardiographic studies performed across the Yale–New Haven Health System (YNHHS) to train deep learning algorithms accounting for variable views and image quality. Next, we deployed the models in retrospective cohorts of patients undergoing POCUS across the emergency departments of YNHHS (internal testing set from Jan 1, 2013, to Dec 31, 2023) and the Mount Sinai Health System (MSHS, external testing set from Jan 1, 2012, to Jan 31, 2024). The primary aim was to develop and validate the performance of an AI-enabled POCUS-adapted approach in identifying hypertrophic cardiomyopathy and transthyretin amyloid cardio myopathy among individuals undergoing real-world POCUS. Acknowledging the expected under-diagnosis of hypertrophic cardiomyopathy and transthyretin amyloid cardiomyopathy, we further addressed two key secondary objectives. First, among patients who had POCUS and were eventually diagnosed with hypertrophic cardio myopathy or transthyretin amyloid cardio myopathy, we examined the time difference between their first positive screen by AI-POCUS and their eventual clinical diagnosis. Second, to examine the prognostic implications of a positive screen among those who were never diagnosed with cardiomyopathy, we assessed the association between the label-specific probabilities at the time of POCUS and mortality (figure 1).

### Key label definitions

For training in YNHHS, we used a composite of diagnostic elements to define disease labels (0=condition not known to be present and 1=condition present). We first identified all individuals in the YNHHS with at least one ICD code suggestive of cardiomyopathy or clinical heart failure (appendix p 5). We then narrowed down to those who had confirmatory cardiac imaging, including cardiac magnetic resonance evidence of hypertrophic cardiomyopathy,^11^ or abnormal nuclear cardiac amyloid testing (eg, Tc99^m^-pyrophosphate) for transthyretin amyloid cardiomyopathy (appendix p 2).^25^ For hypertrophic cardiomyopathy, which often represents a genetic cardiomyopathy with causative variants identified in 40–60% of cases,^11^ we included all available transthoracic echocardiographic and POCUS studies regardless of their timing.^26^ For transthyretin amyloid cardiomyopathy, we defined the time of diagnosis as the time of the positive nuclear cardiac amyloid scan, and, to account for the expected delay between disease onset and diagnosis (reported median of about 12–13 months),^27,28^ we included transthoracic echocardiographic and POCUS studies that were performed up to 12 months before this date (or any time after).

### Development (transthoracic echocardiographic) cohort in the YNHHS

The models were developed using a case–control sample drawn from transthoracic echocardiograms performed across all YNHHS sites (including all five hospitals and affiliated outpatient clinics across Connecticut and Rhode Island, USA; between Jan 1, 2016, and Dec 31, 2022) after excluding any patients known to have had POCUS imaging to prevent subsequent data leakage. All videos during model development were derived from clinical transthoracic echocardiograms conducted by certified sonographers and interpreted by certified cardiologists,^29^ with augmentation of these images to develop models adapted for POCUS. During training, we enriched our sample for cases of severe aortic stenosis (including low-flow low-gradient cases).^30^ This was done to ensure that the model learned to identify it as a separate pathology given its frequent co-occurrence with the labels of interest.^14^ Controls were selected by randomly sampling transthoracic echocardiograms from the same period after excluding any cases of hypertrophic cardiomyopathy, transthyretin amyloid cardiomyopathy, or aortic stenosis, and those with equivocal cardiac magnetic resonance or nuclear imaging results. We used all possible views and randomly split our development cohort at the patient level into a derivation set (training 75% and validation 15%), and transthoracic echocardiogram testing set (10%). Of note, we chose a split sample approach over k-fold cross-validation to balance computational efficiency and due to the size of our training dataset. The validation set was used to track the model’s performance during training and select the best model, whereas the test set was used for final model evaluation. To ensure controls did not include missed cases during training, we excluded studies with a measured interventricular septal thickness of 1·3 cm or more from the controls of our training set. This was not done during validation or testing to ensure reliable model performance assessment.

### Testing (POCUS) cohorts in the YNHHS and the MSHS

The models were tested in retrospective cohorts of individuals who underwent cardiac POCUS across the YNHHS (internal testing; between Jan 1, 2013, and Dec 31, 2023) and the MSHS (external testing; between Jan 1, 2012 and Jan 31, 2024). Studies were performed using compact mid-range ultrasonography systems (ie, Sparq Ultrasound system, Philips Healthcare, Andover, MA, USA in the YNHHS). Here, we excluded participants with end-stage renal disease, a transplanted heart, or prosthetic aortic valve, as well as POCUS studies that exclusively included non-cardiac imaging (ie, lung ultrasonography) with none of the major cardiac views typically captured in a cardiac POCUS (parasternal long-axis, parasternal short-axis at the papillary muscle level, or apical-4-chamber views). In the YNHHS, labels were adjudicated as in the original development cohort (for aortic stenosis, we required a transthoracic echocardiograph showing severe aortic stenosis up to 12 months after the scan), and patient outcomes were extracted through linkage to the Connecticut Death Index (until June 4, 2024). In the MSHS, hypertrophic cardiomyopathy, transthyretin amyloid cardiomyopathy, and aortic stenosis were defined using ICD-10 codes (appendix p 5).

### Automated view characterisation and alignment assessment

Across transthoracic echocardiograph and POCUS studies, we implemented a pipeline that pre-processes echocardiographic videos (appendix pp 2–3).^22^ We applied a validated convolutional neural network that enables video-level classification of 18 standard echocardiographic views by assigning a probability that a given video corresponds to a standard anatomical view.^31^ The highest probability (0–1) defined the most likely view, and was used as a surrogate metric of confidence in the anatomical alignment, with probabilities of 0·5 or higher suggesting high confidence.

### Designing a view-agnostic training pipeline adapted for low-quality acquisitions

We designed a training framework that integrated multiple views (ie, apical, parasternal long, parasternal short, and subcostal views) without annotations and a customised training loss to assign higher weights to low-quality, off-axis videos. We first initialised a 3D-ResNet18 convolutional neural network by using pretrained weights from the Kinetics-400 dataset,^32^ and further modified the output layer of the label to enable multi-label classification for hypertrophic cardiomyopathy, transthyretin amyloid cardiomyopathy, and severe aortic stenosis. To reflect the unique challenges of POCUS, we implemented a range of customisations.

For natural and synthetic data augmentation methods, we trained separate view-specific models for each key view, namely parasternal long-axis, parasternal short-axis, and apical-4-chamber, and all-inclusive, view-naive models trained in pooled datasets that included all parasternal (long and short) and apical views with the classifier blinded to the input view. This method enabled a head-to-head comparison of how view-specific versus view-agnostic approaches generalise to POCUS. We further applied augmentations for variable orientation and off-axis views, including random horizontal flipping and rotation (appendix p 3).

For quality-adjusted weights and loss function, we customised our loss function to favour inference from acquisitions for which the view classifier had lower confidence in assigning a standard view. For this, the sigmoid binary cross entropy loss function was adapted to incorporate label-specific weights to account for rare labels and inverse weighting based on the view alignment probabilities (appendix pp 3–4).^31^

### Model training and inference

All models were trained for a maximum of 30 epochs with early stopping, such that if the mean validation area under the receiver operating characteristic (AUROC) curve in the validation set did not improve for five epochs, training was terminated and the weights from the epoch with maximum validation AUROC curves were used for final evaluation. At the time of inference, we averaged four 16-frame-clip-level predictions to obtain video-level predictions. We analysed the performance of video-level and study-level predictions by averaging label-specific probabilities (between 0 and 1, with higher values suggesting higher confidence that the label is present) from all videos in a study (appendix pp 3–4).

### Model explainability

To assist with explainability, we generated sample saliency maps for the most confident cases using gradient-weighted class activation mapping.^33^ We present examples for each label using the pixel-wise maximum along the temporal axis to capture the most salient spatial regions as a heatmap.

### Statistical analysis

Continuous variables are presented as mean (SD) and median (25th–75th percentile) and compared using Student’s unpaired *t* test or Mann–Whitney *U* test, as appropriate. Categorical variables are summarised as counts (and percentages) and compared across groups using the χ^2^ test. Label-specific thresholds were computed based on the cutoff values that correspond to: the maximal Youden’s J (ie, the sum of the sensitivity and specificity minus one), and 90% sensitivity in the transthoracic echocardiographic test set. Across groups and key subgroups (ie, females *vs* males, individuals without known hypertension, and individuals without known heart failure), metrics of discrimination (ie, AUROC curve) for each label and their difference across models (δ[AUROC]) are provided with 95% CIs from bootstrapping with 1000 replications. We also report sensitivity, specificity, positive predictive value, negative predictive value, the number needed to test (ie, 1 divided by the positive predictive value), and the diagnostic odds ratios (ORs) at 3% prevalence for transthyretin amyloid cardiomyopathy or aortic stenosis, and 1% prevalence for hypertrophic cardiomyopathy, based on their estimated true prevalence in similar cohorts.^4,34^ Based on post hoc calculations, a minimum sample of 5000 observations, with a minimum label prevalence of 0·2%, α of 0·05, and an estimated AUROC curve of ≥0·80, would provide 92·8% power to discriminate cases from controls.^35^

Survival analyses were performed using multivariable Cox regression models for all-cause mortality with each label-specific probability as the independent variable of interest and adjustment for baseline age, sex, hypertension, diabetes, ischaemic heart disease, peripheral arterial disease, and chronic kidney disease. The proportional hazards assumption was assessed and found to be valid based on the weighted (Schoenfeld) residuals method.^36^ For each label, we present two analyses: an analysis of quintiles, and an analysis of the continuous probability using a restricted cubic spline with k=3 knots. We also visualise the unadjusted Kaplan–Meier survival curves compared by the log-rank test. The origin of the analysis is the first POCUS visit for each participant plus a 30-day blanking period, with censoring at the time of death, or the last day with available outcomes data.

All tests were two-sided with a significance level of 0·05 unless specified otherwise. Reporting stands consistent with the TRIPOD-AI statement.^37^ All analyses were performed using Python (version 3.11.2) and R (version 4.2.3). The study was approved by the Yale Institutional Review Board and local Institutional Review Boards, which waived the need for informed consent in the setting of retrospective medical record review.

### Role of the funding source

The funders of the study had no role in study design, data collection, data analysis, data interpretation, or writing of the report.

## Results

The development cohort included 10 702 transthoracic echocardiographic studies with 290 245 videos for 8460 patients across training and validation sets (7621 patients with 9667 studies, mean age was 68·8 [SD 15·3] years, 3694 [48·5%] patients were female, and 3927 [51·5%] were male) and the testing set (839 patients with 1035 studies, mean age of 69·0 [15·7] years, 413 [49·2%] patients were female, and 426 [50·8%] were male; table 1). Across both sets, 46 398 (16·0%) videos corresponded to hypertrophic cardiomyopathy, 8842 (3·0%) to transthyretin amyloid cardiomyopathy, 41 465 (14·3%) to severe aortic stenosis, and 193 952 (66·8%) were used as controls.

The YNHHS POCUS cohort included 39 546 studies from 33 127 patients (mean age 58·9 [SD 20·5] years, 17 276 [52·2%] patients were female, 14 923 [45·0%] were male, and 928 [2·8%] were recorded as unknown) with 78 054 key echocardiographic views (15 751 [20·2%] parasternal long-axis, 52 477 [67·2%] parasternal short-axis, and 9826 [12·6%] apical-4-chamber). Compared with the transthoracic echocardiographic subset, there were relatively more individuals self-identifying as Hispanic (5073 [15·3%] of 33 127 *vs* 539 [6·4%] of 8460, p<0·0001) or Black (8040 [24·3%] *vs* 587 [6·9%], p<0·0001). Reflecting the prevalence of known diagnoses, 279 (0·4%) of these videos were in patients with hyper trophic cardiomyopathy, 172 (0·2%) in patients with transthyretin amyloid cardiomyopathy, and 1130 (1·4%) in patients with severe aortic stenosis.

The MSHS POCUS cohort included 5906 studies from 5624 patients (mean age 56·0 [SD 20·5] years, 1953 [34·7%] patients were female, 2470 [43·9%] were male, and 1201 [21·4%] were recorded as unknown) with 13 796 clips corresponding to key echocardiographic views (5876 parasternal long-axis, 5237 parasternal short-axis, and 2683 apical-4-chamber). Among these, 74 (0·5%) videos were in patients with hypertrophic cardiomyopathy, 32 (0·2%) with transthyretin amyloid cardiomyopathy, and 584 (4·2%) with aortic stenosis.

In a study-level analysis of the held-out testing transthoracic echocardiographic set, our multi-label view-agnostic classifier successfully discriminated hypertrophic cardiomyopathy (AUROC curve 0·95, 95% CI 0·94–0·96) and transthyretin amyloid cardiomyopathy (0·98, 95% CI 0·96–0·99; appendix p 9). Of note, the ability of the classifier to detect hypertrophic cardiomyopathy and transthyretin amyloid cardiomyopathy was independent of the presence of moderate or severe left ventricular hypertrophy (described in the official report of 89 [8·6%] studies with the respective probabilities reaching an AUROC curve of 0·68 [95% CI 0·57–0·67] and 0·61 [0·57–0·65] for discriminating moderate or severe left ventricular hypertrophy from mild or no left ventricular hypertrophy. On video-level comparisons across three key views (parasternal long-axis, parasternal short-axis, and apical-4-chamber), the view-agnostic model outperformed view-specific models (δ[AUROC] of 0·05 [95% CI 0·03–0·07] for hypertrophic cardiomyopathy and 0·03 [0·01–0·06] for transthyretin amyloid cardiomyopathy; appendix p 10). Optimal video-level thresholds for each label based on Youden’s J and 90% sensitivity are presented in the appendix (p 6). Compared with true negatives, false positives had significantly greater left ventricular thickness (for both labels of hypertrophic cardiomyopathy and transthyretin amyloid cardiomyopathy) and worse parameters of diastolic function (for the transthyretin amyloid cardiomyopathy label; appendix p 7).

Compared with the standard transthoracic echocardiographic videos in the YNHHS, the POCUS videos were characterised by significantly lower confidence in view quality and anatomical alignment (median probability for most likely view class of 0·66 [IQR 0·45–0·90] *vs* 0·93 [0·69–1·00]; p <0·0001). On a video-level analysis of the YNHHS emergency department POCUS cohort, the view-agnostic model reached higher discrimination compared with view-specific models for hypertrophic cardiomyopathy (δ[AUROC] of 0·03 [95% CI 0·00–0·06]) and transthyretin amyloid cardiomyopathy (0·15 [0·10–0·21]). Of note, the view-agnostic model’s probabilities showed relative specificity across the different labels (hypertrophic cardiomyopathy, transthyretin amyloid cardiomyopathy, and aortic stenosis) despite their overlapping phenotypes (appendix p 11).

**Figure 2:**
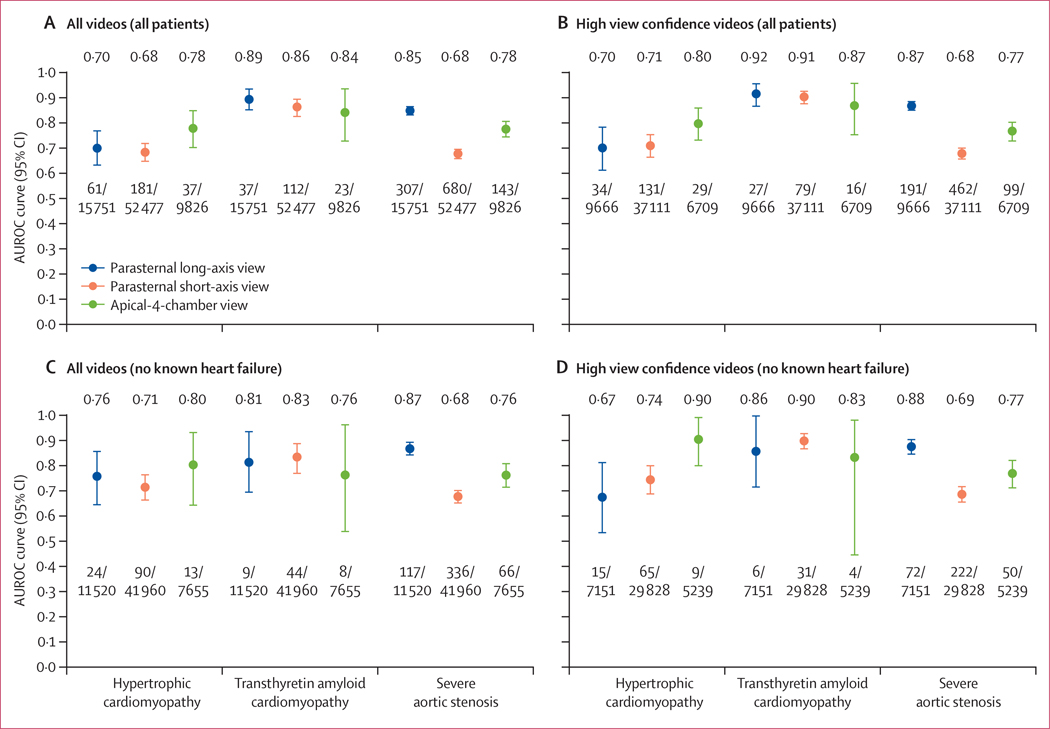
Video-level performance of a view-agnostic multi-label POCUS classifier in the Yale–New Haven Health System Video-level performance (AUROC curve with 95% CIs) for discrimination of hypertrophic cardiomyopathy, transthyretin amyloid cardiomyopathy, and severe aortic stenosis, by deploying a POCUS-adapted model to different echocardiographic views obtained across the emergency departments of the Yale–New Haven Health System. Results for all patients (A, B) and for those without known heart failure at the time of their assessment (C, D) are presented, further stratified by the confidence of the automatic view classifier in detecting the corresponding view (all videos [A, C] *vs* view confidence probability of ≥0·5 [B, D]). The numbers at the bottom of each bar denote the counts of cases out of all eligible video counts in this group. All 95% CIs are derived from bootstrapping with 1000 replications. AUROC=area under the receiver operating characteristic. POCUS=point-of-care ultrasonography.

The diagnostic performance of the AI classifier varied according to the input view, the confidence of the view classifier, and the target population, as summarised in figure 2 and the appendix (p 11), with detailed metrics for selected thresholds summarised in table 2 and the appendix (p 11). For instance, when screening for hypertrophic cardiomyopathy using a single-view apical-4-chamber protocol, the performance of the classifier ranged from 0·800 (95% CI 0·735–0·864) among all patients to 0·903 (0·795–0·981) among those without known heart failure (figure 2), and 0·928 (0·820–0·994) among those without hypertension (appendix p 13). For transthyretin amyloid cardiomyopathy screening using single-view parasternal acquisitions, the classifier achieved an AUROC curve of 0·919 (95% CI 0·863–0·958 for the parasternal long-axis) and 0·907 (0·874–0·932 for the parasternal short axis). Across labels, performance was consistent across males and females (appendix p 14). These findings were replicated in the MSHS cohort, for which our model achieved an AUROC curve of up to 0·890 (95% CI 0·839–0·938) for hypertrophic cardiomyopathy (apical-4-chamber), and 0·994 (0·992–0·996) and 0·972 (0·959–0·983) for parasternal long-axis and parasternal short-axis-based screening of transthyretin amyloid cardiomyopathy, respectively (table 2; appendix p 8).

**Figure 3:**
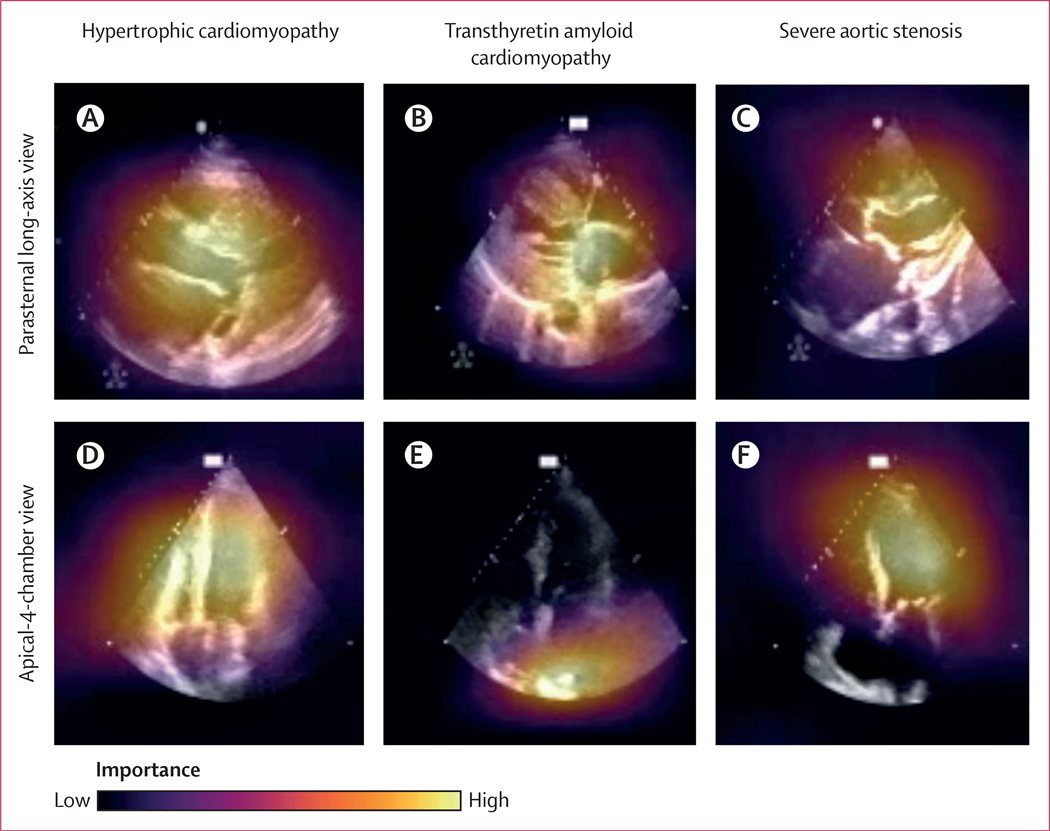
Saliency maps Activation maps for hypertrophic cardiomyopathy (A, B), transthyretin amyloid cardiomyopathy (C, D), and severe aortic stenosis (E, F) across parasternal long-axis and apical-4-chamber views obtained at the point of care in the emergency department. The colour scale denotes the relative importance of different areas, averaged across time, for each individual label.

Representative Gradient-weighted Class Activation Mapping saliency maps for the most confident true positive predictions for each label in the YNHHS POCUS dataset are shown in figure 3. In the examples shown for hypertrophic cardiomyopathy, the signal localised to the left ventricle, whereas for transthyretin amyloid cardiomyopathy, the signal extended to the left atrium. For the reference label of severe aortic stenosis, the focus was on the left ventricle and the aortic valve (when in plane).

We provide sample frames from studies corresponding to the highest and lowest predictions for both cases and controls across all three key views (parasternal long-axis, parasternal short-axis, apical-4-chamber; appendix pp 15–16). These examples showcase the variation in acquisition quality, probe orientation, and presence of noise artifacts.

**Figure 4:**
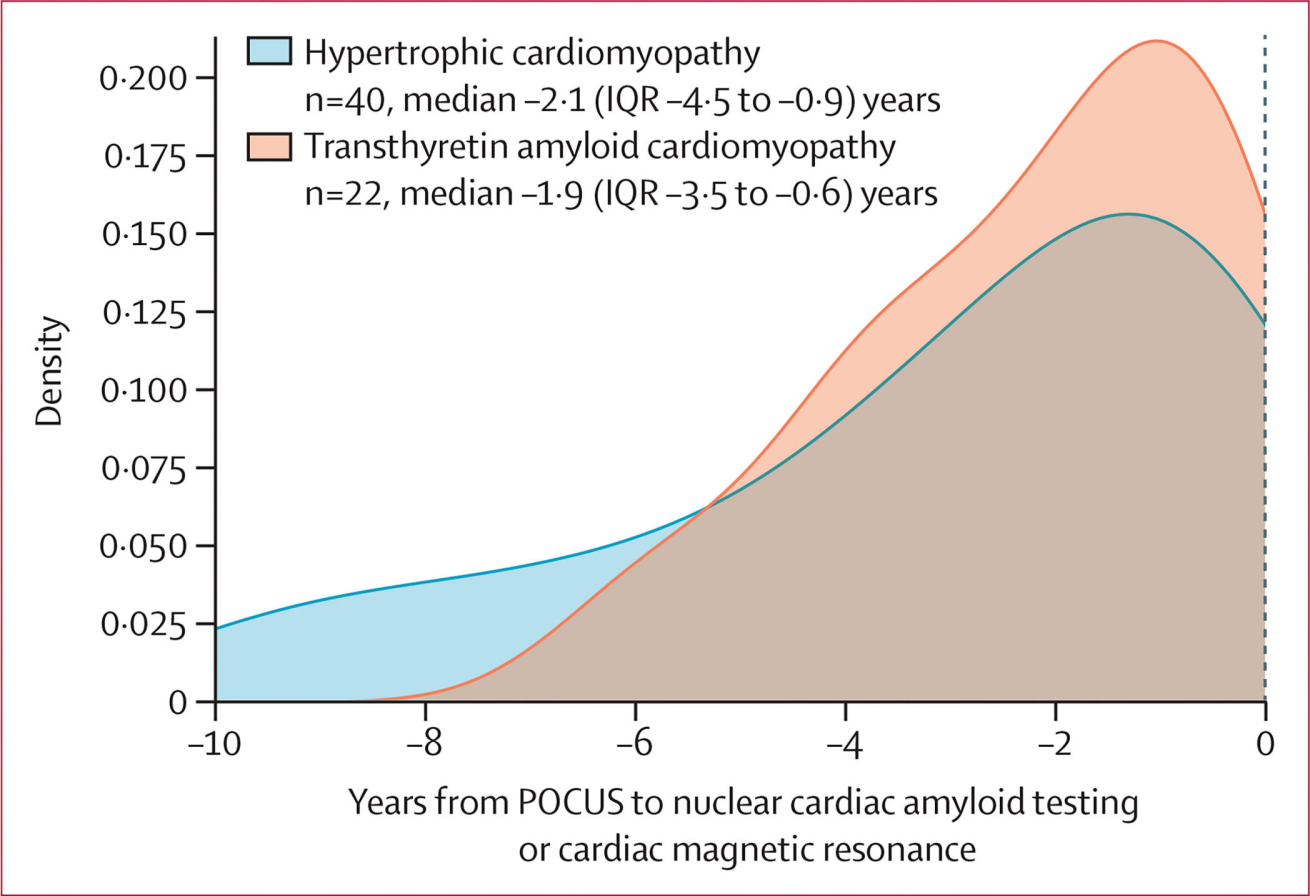
Density plot of time between positive artificial intelligence-POCUS screen and eventual confirmatory testing Density plot summarising the time difference between a positive POCUS screen and confirmatory testing by cardiac magnetic resonance or nuclear cardiac amyloid testing for 40 patients and 23 patients with an eventual diagnosis of hypertrophic cardiomyopathy or transthyretin amyloid cardiomyopathy, respectively. POCUS=point-of-care ultrasonography.

To explore the potential of timely screening using AI-assisted POCUS inference, we estimated that among all patients with hypertrophic cardiomyopathy (n=69) or transthyretin amyloid cardiomyopathy (n=48) who had at least one emergency department POCUS study in the YNHHS (before or after their diagnosis), 40 (58%) and 22 (46%) had a positive screen by POCUS any time before their eventual confirmatory imaging at the 90% sensitivity thresholds, respectively. Among these participants, the median time between the first positive AI-POCUS and cardiac magnetic resonance or cardiac scintigraphy was 2·1 (IQR 0·9–4·5) and 1·9 (0·6–3·5) years for hypertrophic cardiomyopathy and transthyretin amyloid cardiomyopathy, respectively (figure 4).

**Figure 5:**
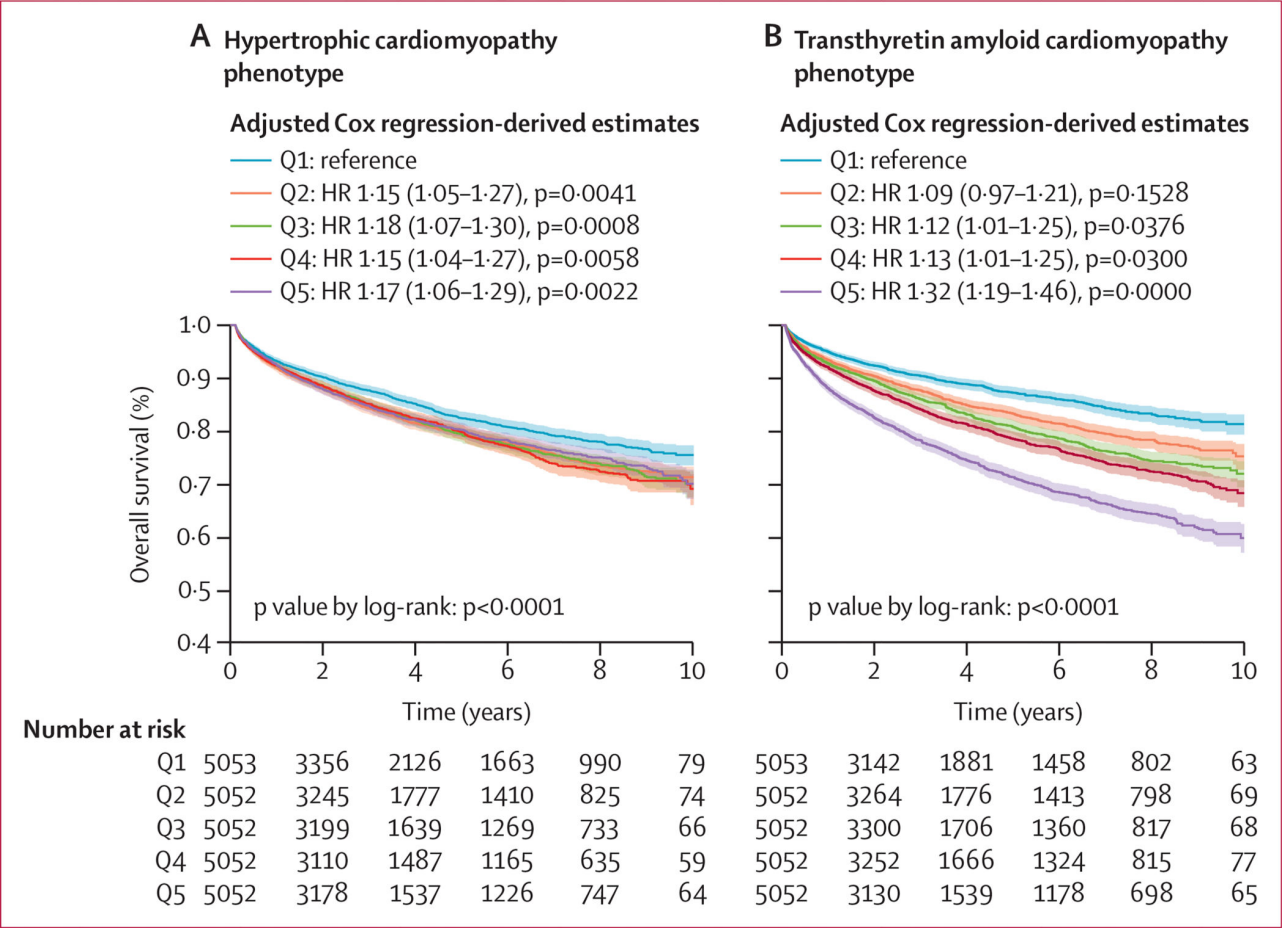
Hypertrophic cardiomyopathy and transthyretin amyloid cardiomyopathy-specific probabilities and overall survival among individuals without documented cardiomyopathy in the YNHHS Kaplan–Meier survival curves across quintiles (Q1–5) of the probabilities for hypertrophic cardiomyopathy (A) and transthyretin amyloid cardiomyopathy (B) on point-of-care ultrasonography in the YNHHS. Results are presented for n=25 261 eligible individuals who were never diagnosed with cardiomyopathy during the follow-up period (median of 2·8 [IQR 1·2–6·4] years). We report the p values for comparison of the Kaplan–Meier curves, and also Cox regression-derived HRs (95% CIs) adjusted for age, sex, hypertension, diabetes, ischaemic heart disease, chronic kidney disease, and peripheral arterial disease. HR=hazard ratio. Q=quintile. YNHHS=Yale–New Haven Health System.

To address the prognostic implications of the AI-POCUS phenotypes, we explored the association between AI-POCUS-derived probabilities and all-cause mortality. Of the 33 127 individuals, 31 860 (96·2%) records were linked to the state death index. Among 25 261 participants (mean age 57·1 [SD 20·2] years; 14 011 [55·5%] females and 11 250 [44·5%] males) without cardiomyopathy followed up over 2 ·8 (IQR 1·2–6·4) years there were 4219 (16·7%) deaths. A hypertrophic cardiomyopathy or transthyretin amyloid cardiomyopathy-like phenotype in the highest (*vs* lowest) respective quintile conferred a 17% (adjusted hazard ratio 1·17, 95% CI 1·06–1·29; p=0·0022 for hypertrophic cardiomyopathy) and 32% higher adjusted risk of mortality (1·32, 95% CI 1·19–1·46]; p<0·0001 for transthyretin amyloid cardiomyopathy), respectively (figure 5, appendix p 17).

## Discussion

We show that an AI algorithm adapted for low-quality cardiac POCUS acquisitions and off-axis views can reliably identify under-diagnosed cardiomyopathies. Our findings suggest a novel role for AI-augmented POCUS interpretation in the opportunistic screening of cardiomyopathies, expanding the scope of a common and accessible imaging modality that is increasingly available across primary care, emergency medicine, and resource-limited health-care settings.^24^ To achieve this, our approach prompts video-based models to adjust to the unique challenges of handheld cardiac ultrasonography. We show the generalisability and scalability of this method across video-level analyses of more than 90 000 distinct POCUS videos acquired across the emergency departments of two large health systems by evaluating their ability to discriminate between hypertrophic cardiomyopathy and transthyretin amyloid cardiomyopathy. We further provide evidence suggesting that AI-POCUS could enable early diagnosis and that these AI-defined phenotypes have prognostic implications, flagging groups without known cardiomyopathy at high risk of adverse outcomes.

Overall, our work is both methodologically and clinically innovative. On the methodological front, we describe a scheme that boosts the performance of AI tools when deployed to real-world POCUS studies by explicitly addressing POCUS-specific challenges as part of the model development process. We show that by incorporating differential weights based on view confidence, along with view-naive and synthetic augmentation techniques, our models generalise to off-axis POCUS acquisitions of varying quality. On the clinical front, our work could promote equitable access at the first point of care.^18^ In our study, we found that the proportion of Black and Hispanic individuals was nearly three times higher in the POCUS cohort than in the transthoracic echocardiographic cohort. This highlights the potential to leverage clinical workflows, particularly for conditions such as hypertrophic cardiomyopathy and transthyretin amyloid cardiomyopathy that benefit from early detection and risk stratification, yet remain under-diagnosed.^16,17,26^ Moreover, our work suggests a path towards efficient and scalable targeted screening for communities at high-risk that could be performed using low-cost equipment and abbreviated protocols by novice operators.^3,24^ Notably, positive signatures might exist several years before clinical diagnosis, and even among those who are never diagnosed with cardiomyopathy, high AI-defined probabilities are associated with worse long-term outcomes. Together, these highlight the potential for reducing the time from first contact to clinical diagnosis, while also reducing the rates of mis-diagnosis or under-diagnosis of treatable cardiomyopathies.

Moving forward, we envision that AI-guided POCUS screening could expand our ability to identify individuals missed through traditional care pathways. This approach could be implemented both retrospectively through systematic screening of imaging repositories, or through prospective integration at the point of care. Systems could choose to flexibly calibrate the approach and tune probability thresholds to optimise sensitivity or specificity depending on their local patient population demographics and proposed clinical or research use (ie, eligibility screening for inclusion in clinical trials). The approach is vendor-agnostic, and offers an interoperable, end-to-end pipeline that automates the processing of raw data types (ie, Digital Imaging and Communications in Medicine [more commonly referred to as DICOM]) and all required inference steps, including multi-label classification for all conditions of interest.

Certain limitations merit consideration. First, our study was a retrospective analysis of clinically indicated scans across two large health systems. However, these represent an appropriate setting for opportunistic screening. Second, our evaluation of model performance is challenged by the known under-diagnosis of hyper trophic cardiomyopathy and transthyretin amyloid cardiomyopathy, with several cases likely flagged as false positives, a hypothesis supported by the observed lead time of diagnosis seen through AI-POCUS and the high rate of adverse outcomes of false positives. However, the diagnostic performance and lead time need to be investigated in prospectively planned studies. Third, POCUS studies were not directly used for model training, and the challenges of point-of-care acquisition were introduced through natural and synthetic augmentations. This enabled the use of a large well annotated dataset of clinical echocardiograms. Future studies will leverage expanding POCUS libraries for in-domain finetuning of these models. Fourth, our model is not intended to inform acute management in the emergency department, but rather to provide an additional layer of support for the opportunistic screening and timely risk stratification of individuals at risk of hypertrophic cardiomyopathy or transthyretin amyloid cardiomyopathy. Fifth, saliency maps only describe isolated cases and are known to perform poorly for complex phenotypes.^38^ Sixth, the outcomes analysis is limited to the YNHHS set given access to the local state death, with about 4% of the eligible individuals excluded due to insufficient information. Moreover, we cannot exclude unintended confounding given the retrospective and observational nature of these associations. Finally, we did not query myocardial biopsy reports, which, along with our strict definitions, could have resulted in low prevalence for our labels. However, diagnoses in the MSHS were defined based on ICD codes, thus supporting the generalisability of the model.

We show that AI models adapted for use with POCUS can reliably identify hypertrophic cardiomyopathy and transthyretin amyloid cardiomyopathy at the point of care. Our results support a scalable and inexpensive screening approach that uses automated AI-based inference on an accessible and portable modality to identify conditions that typically require advanced multimodality imaging, with the potential for early diagnosis, risk stratification, and improved outcomes.

## Supplementary Material

1

## Figures and Tables

**Table 1:** Summary of cohort characteristics

	Development (transthoracic echocardiographic) cohort		Testing emergency department cohorts (point-of-care ultrasonography)	
	Training and validation set (n=7621)	Testing set (n=839)	Yale–New Haven Health System (n=33 127)	Mount Sinai Health System* (n=5624)
Number of unique studies	9667	1035	39 546	5906
Number of unique videos	261 756	28 489	78 054	13 796
Parasternal long-axis view	30 556 (11·7)	3283 (11·5)	15 751 (20·2)	5876 (42·6)
Parasternal short-axis view (papillary muscle level)	31 779 (12·2)	3313 (11·6)	52 477 (67·2)	5237 (38·0)
Apical 4-chamber view	25 528 (9·8)	2649 (9·3)	9826 (12·6)	2683 (19·4)
Participant-level demographics				
Age at the time of scan, years	68·8 (15·3)	69·0 (15·7)	58·9 (20·5)	56·0 (20·5)
Sex				
Female	3694 (48·5%)	413 (49·2%)	17 276 (52·2%)	1953 (34·7%)
Male	3927 (51·5%)	426 (50·8%)	14 923 (45·0%)	2470 (43·9%)
Unknown	··	··	928 (2·8%)	1201 (21·4%)
Hispanic ethnicity				
Hispanic	489 (6·4%)	50 (6·0%)	5073 (15·3%)	1094 (19·5%)
Non-Hispanic	6806 (89·3%)	762 (90·8%)	26 796 (80·9%)	2656 (47·2%)
Unknown	326 (4·3%)	27 (3·2%)	1258 (3·8%)	1874 (33·3%)
Race				
Asian	102 (1·3%)	14 (1·7%)	616 (1·9%)	189 (3·4%)
Black or African American	527 (6·9%)	60 (7·2%)	8040 (24·3%)	1385 (24·6%)
White	6353 (83·4%)	701 (83·6%)	19 535 (59·0%)	1215 (21·6%)
Other or unknown	639 (8·4%)	64 (7·6%)	4936 (14·9%)	2835 (50·4%)
Video-level labels				
Hypertrophic cardiomyopathy	41 602 (15·9%)	4796 (16·8%)	279 (0·4%)	74 (0·5%)
Transthyretin amyloid cardiomyopathy	7920 (3·0%)	922 (3·2%)	172 (0·2%)	32 (0·2%)
Aortic stenosis†	37 270 (14·%2)	4195 (14·7%)	1130 (1·4%)	584 (4·2%)

Data are n, n (%) or mean (SD).

* Diagnoses in the Mount Sinai Health System cohort were defined based on ICD codes.

† Severe aortic stenosis by echocardiography in the Yale–New Haven Health System dataset, or any aortic stenosis by diagnosis codes in the Mount Sinai Health System.

**Table 2:** Performance of single-view point-of-care ultrasonography screening strategies for hypertrophic cardiomyopathy and transthyretin amyloid cardiomyopathy

	Cohort	Area under the receiver operating characteristic curve (95% CI)	Sensitivity (95% CI)	Specificity (95% CI)	Expected positive predictive value*	Expected negative predictive value*	Positive likelihood ratio	Negative likelihood ratio	Diagnostic odds ratio	Number needed to test
**All studies—transthyretin amyloid cardiomyopathy**										
Parasternal long-axis view	Yale–New Haven Health System	0·894 (0·856–0·931)	0·811 (0·675–0·935)	0·765 (0·757–0·771)	0·096	0·992	3·451	0·247	14·0	10
Parasternal long-axis view	Mount Sinai Hospital System	0·994 (0·992–0·996)	1·000 (NA)	0·794 (0·784–0·804)	0·131	1·000	4·854	0·000	Infinity	8
Parasternal short-axis view (papillary muscle)	Yale–New Haven Health System	0·864 (0·832–0·896)	0·679 (0·596–0·757)	0·862 (0·859–0·865)	0·132	0·989	4·920	0·372	13·2	8
Parasternal short-axis view (papillary muscle)	Mount Sinai Hospital System	0·973 (0·959–0·984)	1·000 (NA)	0·765 (0·754–0·776)	0·116	1·000	4·255	0·000	Infinity	9
**All studies—hypertrophic cardiomyopathy** †										
Apical 4-chamber	Yale–New Haven Health System	0·800 (0·669–0·935)	0·538 (0·300–0·774)	0·902 (0·894–0·907)	0·053	0·995	5·490	0·512	10·7	19
Apical 4-chamber	Mount Sinai Hospital System	0·891 (0·838–0·936)	0·909 (0·772–1·0)	0·726 (0·710–0·743)	0·032	0·999	3·318	0·125	26·5	31
**High confidence—transthyretin amyloid cardiomyopathy**										
Parasternal long-axis view	Yale–New Haven Health System	0·919 (0·863–0·958)	0·852 (0·735–0·962)	0·770 (0·763–0·776)	0·103	0·994	3·704	0·192	19·3	10
Parasternal long-axis view	Mount Sinai Hospital System	0·994 (0·992–0·996)	1·000 (NA)	0·802 (0·791–0·818)	0·135	1·000	5·051	0·000	Infinity	7
Parasternal short-axis view (papillary muscle)	Yale–New Haven Health System	0·907 (0·874–0·932)	0·734 (0·635–0·818)	0·874 (0·871–0·878)	0·153	0·991	5·825	0·304	19·2	7
Parasternal short-axis view (papillary muscle)	Mount Sinai Hospital System	0·972 (0·959–0·983)	1·000 (NA)	0·765 (0·753–0·777)	0·116	1·000	4·255	0·000	Infinity	9
**High confidence—hypertrophic cardiomyopathy** †										
Apical 4-chamber	Yale–New Haven Health System	0·903 (0·795–0·981)	0·667 (0·316–0·905)	0·911 (0·903–0·918)	0·070	0·996	7·494	0·366	20·5	14
Apical 4-chamber	Mount Sinai Hospital System	0·890 (0·839–0·938)	0·900 (0·737–1·0)	0·734 (0·716–0·751)	0·033	0·999	3·383	0·136	24·9	30

Threshold-dependent metrics are presented at cutoffs that maximise Youden’s J. NA=not applicable.

* Positive predictive value and negative predictive value are reported at a simulated prevalence of 3% for transthyretin amyloid cardiomyopathy (including patients with heart failure) and 1% for hypertrophic cardiomyopathy.

† Screening for hypertrophic cardiomyopathy was done in a population without known heart failure in the Yale–New Haven Health System cohort (per previous echocardiography or diagnosis codes).

## Data Availability

The underlying videos might contain identifiable information and cannot be released at this time. The analytical code and executable or containerised versions of the model for direct inference can be made available upon request to the corresponding author within the context of a research collaboration.

## Research in context

### Evidence before this study

Point-of-care ultrasonography (POCUS) can support clinical decision making at the point of care as a direct extension of the physical examination. POCUS has benefited from the increasing availability of portable and smartphone-adapted probes and even artificial intelligence (AI) solutions that can assist novices in acquiring basic views. However, the diagnostic and prognostic inference from POCUS acquisitions is often limited by the short acquisition duration, suboptimal scanning conditions, and limited experience in identifying subtle pathology that goes beyond the acute indication for the study. Solutions have shown the potential of AI-augmented phenotyping in identifying traditionally under-diagnosed cardiomyopathies on standard transthoracic echocardiograms performed by expert operators with strict protocols. However, these are not optimised for opportunistic screening using videos derived from typically lower-quality POCUS studies. Given the widespread use of POCUS across communities, ambulatory clinics, emergency departments, and inpatient settings, there is an opportunity to leverage this technology for diagnostic and prognostic inference, especially for traditionally under-recognised cardiomyopathies, such as hypertrophic cardiomyopathy or transthyretin amyloid cardiomyopathy, which could benefit from timely referral for specialised care.

### Added value of this study

We present a multi-label, view-agnostic, video-based deep learning algorithm adapted for POCUS use, which can reliably discriminate cases of transthyretin amyloid cardiomyopathy and hypertrophic cardiomyopathy versus controls. The model’s performance was examined across more than 90 000 unique POCUS videos acquired over a decade across emergency departments affiliated with two large and diverse health systems. The model benefits from customised training that emphasises low-quality acquisitions as well as off-axis, nontraditional views, outperforming view-specific algorithms and approaching the performance of standard transthoracic echocardiographic algorithms using single POCUS videos as the sole input. We further provide evidence that, among reported controls who were never diagnosed with cardiomyopathy, higher probabilities for hypertrophic cardiomyopathy or transthyretin amyloid cardiomyopathy-like phenotypes are associated with worse long-term survival, suggesting possible under-diagnosis with prognostic implications. Finally, among confirmed cases with previously available POCUS imaging, positive AI-POCUS screens were seen at a median of 2 years before eventual confirmatory testing, highlighting an untapped potential for timely diagnosis through opportunistic screening.

### Implications of all the available evidence

We define an AI framework with excellent performance in the automated detection of under-diagnosed yet treatable cardiomyopathies. This strategy could enable scalable screening, detecting these disorders years before their clinical recognition, thus improving the diagnostic and prognostic inference of POCUS imaging in clinical practice.
